# The lateral Jobe test: A more reliable method of diagnosing rotator cuff tears

**DOI:** 10.4103/0973-6042.70822

**Published:** 2010

**Authors:** John Joseph Gillooly, Ramiah Chidambaram, Daniel Mok

**Affiliations:** Shoulder Unit, Department of Trauma and Orthopedic Surgery, Epsom and St. Helier NHS Trust, UK

**Keywords:** Clinical examination, lateral Jobe test, rotator cuff tears

## Abstract

**Purpose:**

The most reliable clinical investigations to diagnose rotator cuff tears reported in the literature is a triad of weakness on resisted external rotation, pain on impingement, and weakness on supraspinatus testing, or a combination of two of the above in a patient over 60 years of age. We present a simple new clinical test “The lateral Jobe Test” and compare it to these combined tests. The lateral Jobe test is performed with the patient’s shoulder abducted 90° in the coronal plane and internally rotated so that with the elbows flexed 90° the fingers point inferiorly and thumbs medially. A positive test is pain or weakness on resisting an inferiorly directed force applied to the distal arms or an inability to perform the test.

**Materials and Methods:**

A consecutive series of 175 patients undergoing shoulder arthroscopy were reviewed prospectively and examined by two independent orthopedic surgeons blinded to the diagnosis. The results of the clinical tests were validated against arthroscopic findings.

**Results:**

The lateral Jobe test had a significantly higher sensitivity (81 vs. 58%) than the combined tests. The specificity of both was similar at 89 and 88%, respectively.

**Conslusion:**

The lateral Jobe test is a simple single test which can help in the clinical diagnosis of rotator cuff tears.

**Level of Evidence:**

Level IIb

## INTRODUCTION

Rotator cuff tears comprise almost 50% of shoulder injuries, and cadaveric studies have estimated their incidence at 30% in those over the age of 60.[1] Clinical evaluation remains the mainstay of initial diagnosis however these injuries can sometimes pose a diagnostic challenge. A review of 23[2] clinical tests reported that the most reliable method of confirming rotator cuff tears was either a triad of weakness in resisted external rotation, pain on impingement, and weakness on supraspinatus testing (Jobe’s test)[3] or a combination of two of the above in a patient aged 60 or older.

We present a simple new clinical test to diagnose rotator cuff tears “The Lateral Jobe Test” and compare it to the published literature.

**Figure 1:**
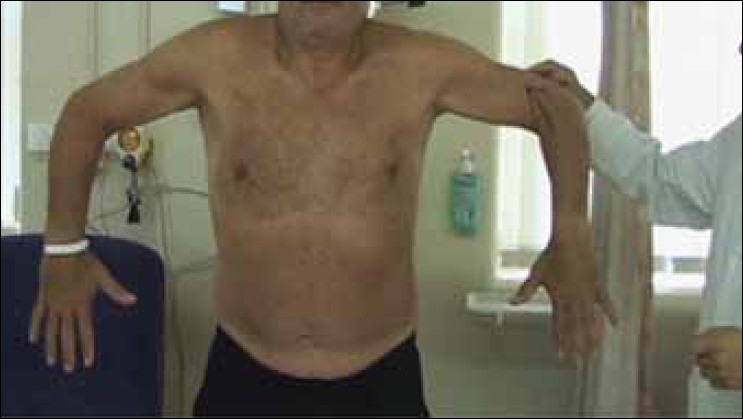
A patient performing the lateral Jobe test

Dr. Joe De Beer in Capetown, South Africa, first demonstrated the lateral Jobe test to the senior author in 2003. It is performed by having the patient abduct their shoulder to 90° in the coronal plane with the elbows flexed 90° and the shoulders internally rotated so that the fingers point inferiorly and the thumbs are directed medially, [Figure 1]. A positive test consists of pain or weakness on resisting an inferiorly directed force applied to the distal arm while in this position or an inability to perform the test.

## MATERIALS AND METHODS

Between September 2006 and January 2007, a consecutive series of 175 patients undergoing arthroscopy for painful shoulder were reviewed prospectively. Their average age was 53 years (range 17-83). There were 97 males and 78 females. Those with either a previous fracture or surgery to the ipsilateral shoulder were excluded. The duration of symptoms was noted. All patients were examined, preoperatively and independently by two senior orthopedic surgeons blinded to the preoperative diagnosis using four tests. These were the lateral Jobe test, the Jobe supraspinatus test, strength in external rotation when compared to the normal side, and impingement testing. A consensus review was taken and the clinical tests were then validated postoperatively against the arthroscopic findings. The final results were assessed for sensitivity, specificity, negative predictive value, and positive predictive value.

## RESULTS

We referred to the supraspinatus test, strength in external rotation, and impingment test as the combined tests, and a positive result for the combined tests was taken as weakness on supraspinatus testing, weakness in external rotation and pain on impingement or a combination of two of these and an age greater than 60 years. The results are given in Table 1.

**Table 1:** Test results

	True positive	True negative	False positive	False negative
Lateral Jobe	83	65	8	19
Combined tests	59	64	9	43

102 of the 175 patients (58%) had rotator a cuff tear confirmed arthroscopically. The lateral Jobe test had a significantly higher sensitivity 81% (95% confidence interval or CI 72-88) than the combined tests 57% (CI 48-67).

Both the lateral Jobe test and the combined tests had a similarly high specificity: 89% (CI 79-95) and 88% (CI 77-94), respectively. The lateral Jobe test did have a higher positive predictive value than the combined tests 91% vs 87% and also a higher negative predictive value than the combined tests 77% vs 60% although neither of these differences was statistically significant.

The arthroscopic findings of the eight false positive lateral Jobe tests were: 5 calcific tendonitis, 1 frozen shoulder, 1 scuffed supraspinatus tendon, and 1 normal shoulder.

The arthroscopic findings of the 19 false negative lateral Jobe tests were: 12 full thickness supraspinatus tears, 5 partial thickness supraspinatus tears, and 2 massive tears.

The average duration of patients’ symptoms before presentation was 15 months. All 18 false negative Lateral Jobe tests had symptom durations that were greater than 2 years.

The type and number of rotator cuff tears noted on arthroscopy were: 71 supraspinatus tears, 15 massive tears, 10 partial thickness supraspinatus tears, 4 supraspinatus and subscapularis tears, 1 supraspinatus and infraspinatus tear and 1 subscapularis tear.

## DISCUSSION

An NHS Health and Technology Assessment review of the available literature on the clinical diagnosis of rotator cuff tears in 2003 stated that “there was no conclusive evidence for any single test that can conclusively diagnose rotator cuff disorders.”[4] Lyons and Tomlinson[5] in their study on 42 preoperative rotator cuff tear patients noted that testing for weakness in supraspinatus and infraspiantus as well as palpation of the humeral head for a cuff defect had a 91% sensitivity and a 75% specificity. That study was flawed because their inclusion criteria were patients with a previously clinically diagnosed rotator cuff tear. Analysis of the results of examination on that patient group would naturally tend toward a more positive result.

Murrell and Walton[3] performed a prospective study of 400 patients and compared the results of 23 clinical tests with the findings at subsequent shoulder arthroscopy. They found that the combined tests had a 98% specificity for the diagnosis of rotator cuff tears. Our study confirmed a high specificity for the combined tests but at 88% it fell short of the quoted figure but was of a similar level to the specificity for the lateral Jobe test 89%. 75% of the false positive lateral Jobe tests were due to either calcific tendonitis or frozen shoulder. Clinical examination has been shown to have a high sensitivity and specificity for frozen shoulder[6] and plain X-ray demonstrates the majority of deposits in calcific tendonitis.[7] We therefore recommend that frozen shoulder and calcific tendonitis are out-ruled using these techniques to improve the specificity of the lateral Jobe test. Another condition that, although not found in this study, may result in false positive results is contracture of the posterior capsule of the shoulder. This condition causes pain and a loss of internal rotation and has been associated with rotator cuff injury.[8] Care should be taken in the evaluation of all positive patients, to measure and compare the degree of internal rotation of the shoulder to ensure this condition is not missed, and thereby reduce possible false positive results.

Importantly at 81% the sensitivity of the lateral Jobe test was significantly higher than the 57% sensitivity of the combined tests. All of the false negative lateral Jobe tests were in patients who had symptom durations longer than 24 months, well above the average duration of 15 months. Although this implied that the sensitivity of this test might deteriorate over the course of time it also indicated that the sensitivity of the lateral Jobe test would be greater if performed on patients with symptom durations of less than 2 years. A biomechanical explanation for these false negative results is provided by the concept of muscular compensation. It has been shown that in rotator cuff tears a variety of muscles including the deltoid, pectoralis major, and latissimus dorsi adapt to compensate for the loss of the rotator cuff in massive tears.[9] In isolated supraspinatus tears, the infraspinatus and teres minor have also been shown to contribute to the compensatory mechanism and nearly two thirds of patients with full thickness rotator cuff tears treated non-operatively experience functional improvement at 18 months.[10] The time-related deterioration of the sensitivity of this test implies that as compensation occurs and function returns, this test becomes more unreliable.

To medical practitioners with little orthopedic training this simple test if positive may increase the suspicion of a rotator cuff tear and result in a more prompt referral to a shoulder surgeon. In the outpatient department the high sensitivity of this test in conjunction with a thorough history and examination may help provide a more reliable clinical diagnosis of rotator cuff tears and reduce the need for further imaging.

We conclude that the lateral Jobe test is a simple, new technique, which can improve the clinical diagnosis of rotator cuff tears.
